# Lessons from a crisis—opportunities for lasting public health change from the COVID-19 pandemic

**DOI:** 10.3389/fpubh.2022.893871

**Published:** 2022-10-19

**Authors:** Jaskanwal Deep Singh Sara

**Affiliations:** Department of Cardiovascular Medicine, Mayo Clinic, Rochester, MN, United States

**Keywords:** COVID-19, vaccination, medical education, telemedicine, pandemic (COVID-19), collaboration

## Abstract

The coronavirus 2019 (COVID-19) global pandemic has wrought hardship and disrupted lives across all strata of humanity, giving rise to a variety of social, psychological, and medical challenges to individuals in almost every country in the world. Yet for all the difficulties the pandemic has inflicted, it has forced us to examine previously accepted practices at home, work, and society more broadly and has led to innovative changes in the way we communicate and collaborate. These novel approaches to contemporary challenges were devised primarily to allow continued productivity despite the need for social distancing, but have offered secondary advantages that could provide society with lasting benefits. In the following review, we outline three aspects of working life and public health which could experience lasting improvement on the back of lessons learnt from the current crisis.

## Introduction

The coronavirus 2019 (COVID-19) global pandemic has unambiguously wrought hardship and disrupted lives across all strata of humanity. In doing so, the pandemic has given rise to a bleak chapter not to be forgotten by any of us anytime soon. COVID-19 has spread to almost every country in the globe with almost 400 million cases and more than 5.5 million deaths worldwide (1). The highest number of confirmed cases and deaths in the United States was over 75 million and 890,000, respectively; at the time of this writing, numbers made all the more staggering when considering the fact that the first year of the pandemic saw the virus killing more people in the United States than stroke, influenza, suicides, and car crashes combined (1). Moreover, neither world economies nor individual businesses have been able to escape its grasp, with unemployment reaching 15% at the height of the first wave, prompting the government in the United States to enact six major relief bills amounting to more than $5 trillion (1). Undoubtedly, the pandemic has affected individuals at all levels of society across the world, bringing with it a host of stressors including job loss or job and income insecurity, illness and deaths of loved ones, and social isolation and loneliness. While the longer-term implications of the COVID-19 pandemic are yet to be realized, it is likely that the health and economic consequences faced by individuals and families across the world during this time will be vast.

So, naturally, who would not want to return to the “good old days”—a simpler, safer, and more familiar time. In many ways, the return leg of that journey is well underway. The vaccination campaign in the United States commenced in December 2020, with more than 50% of the population in the country fully vaccinated (1). Lockdown policies and social distancing restrictions have largely been lifted, and minds are concentrating on a potential return to a semblance of normalcy this year. This then begs the question whether our existing concept of “normal” henceforth should be revised. Are there some aspects of pandemic life that we should retain? Did any good come of this time, or was all our suffering for nothing? Winston Churchill was purported to say “never let a good crisis go to waste” in the early days of the Second World War. Indeed, the former British Prime Minister was referring to early events of what in its own right was a terrifying chapter of human history. For all the misfortunes the pandemic has inflicted, it has forced us to examine previously accepted practices at home, work, and society more broadly and has led to innovative changes in the way we communicate and collaborate. Outside the usual scope of workplace navigation, these changes were devised primarily to allow continued productivity despite the need for social distancing, but through serendipity have yielded secondary advantages that we should be cautious about disregarding in our eagerness to return to familiarity.

## Tele-healthcare

The pandemic has led to an unparalleled shift from in-person care to remote visits (2), facilitated in part by changes in reimbursement policies. The use of telemedicine has increased gradually over recent years although there has been a sharp surge in its uptake during the pandemic, laying the basis for remote clinics forming a larger and more permanent aspect of healthcare delivery (3–5). Studies have shown the benefits of remotely delivered healthcare through event monitors, smart devices, and wearables on various disease processes including hypertension and heart failure (6, 7). Indeed, while traditional healthcare models require in-person evaluation with potentially lengthy visits and costly testing, telemedicine holds the promise of offering simple, inexpensive, and non-invasive methods of evaluation that are undertaken remotely from healthcare providers, which may therefore reduce risk of transmission of diseases between patients and providers. Further, studies have demonstrated that telemedicine has the potential to improve care for patients (7, 8). Nevertheless, the implications of such a large-scale transition to remote healthcare on real-life clinical practice patterns as well as patient care and outcomes are still to be determined. This would be particularly important given the lack of established guidelines outlining best practice for remote care, the potential for unintended consequences that include those created by the so-called digital divide whereby specific patient groups such as those who are older, from racial and ethnic minority groups, and with more comorbidities might be less able to use remote care through lack of access to the Internet and technology literacy or through a lack of physical examinations resulting in an excess use of unnecessary testing and overprescribing medications. Surprisingly, in a recent study looking at a large number of ambulatory cardiology visits the authors found a significantly higher use of remote cardiology clinic visits among Asian, Black, and Hispanic individuals and those with cardiovascular comorbidities (9). Less surprisingly, they found that patients with private insurance, a proxy for high socioeconomic status, made up a larger proportion of both video and telephone visits. This was consistent with another study of clinics serving low-income individuals that reported a decline in overall patient visits after switching to a telehealth model mainly due to a lack of access to video visits for low-income populations (2). They also demonstrated a stepwise reduction in the ordering frequency of both diagnostic tests and prescription medications when comparing pre-COVID with COVID-era in-person and COVID-era video and COVID-era telephone visits (9), which was all the more remarkable given patients seen by remote visits had more cardiovascular comorbidities and were therefore more likely to require guideline-recommended medical therapies. A variety of explanations may be postulated for these findings. First, studies in the press have focused attention on the increased risk of COVID-19 infection in the elderly, those from ethnic minority backgrounds, and those with cardiovascular comorbidities. This then has the potential for convincing such patients, as well as their clinicians, to differently perceive the risk of attending face-to-face visits and to instead elect to pursue telehealth options (10). Second, older patients, those from ethnic minority backgrounds, or those with more medical comorbidities may find remote visits more appealing because they are relatively less able to access in-person visits due to greater barriers to transportation or scheduling (11). Indeed, higher proportions of individuals from ethnic minority backgrounds work “essential jobs” and so may be less able to take time off from work to travel to in-person visits. Third, some of the decreased testing could similarly be explained by reduced access, as much medical testing is typically undertaken at the same facility and at the same time as in-person clinic visits. Fourth, differences in patterns of ordering tests and in turn prescribing medications may simply be associated with the inherent limitations in understanding each patient's clinical picture when using remote care due to a lack of physical examination and decreased clarity in communication. Prompting for testing is often cued by examination findings, while starting and titrating medication is often directed by the results of laboratory testing. What effects these changes have on longer-term patient outcomes as well as on the structure of clinical practices going forward remain to be seen and will require further follow-up studies after the pandemic has waned. Nonetheless, the fact that a substantial proportion of clinical care in future will be delivered through telehealth provides numerous important opportunities in the efficacy, access, and cost of healthcare that may be best implemented when hybridized with and used as an adjunct to existing in-person practice models.

An important point worth highlighting is that by ensuring the timely and affordable provision of healthcare services, telemedicine is particularly advantageous for developing countries. That said, special consideration should be given to the challenges of making telemedicine an ethical and secure mode delivery of medical care that is accessible to all (12). This could include greater standardization in remote healthcare delivery protocols including the development of guidelines outlining best practices; systematic evaluation of telemedicine practice models to assess their feasibility, safety, and efficacy; large prospective clinical trials evaluating clinical protocols delivered using telemedicine that include diverse populations from high-, middle-, and low-income countries to ensure clinical outcomes are at least non-inferior to those provided by established in-person healthcare models with comparative cost–benefit analyses; establishing the role of and creating guidelines for regulatory agencies and insurance companies as well as private companies that may collaborate with healthcare groups to build telemedicine infrastructure; and instituting robust and standardized measures to safeguard individual privacy and data protection.

## Remote working and education

Government mandates for social distancing and limiting the number of people attending in-person indoor public gatherings have led to a surge in the so-called working from home economy in which 42% of the labor force in the United States worked from home, while 26% worked on business premises, the majority of whom were essential service workers (13). Further, the greatly enlarged proportion of home workers accounts for more than two-thirds of the country's economic activity in terms of gross domestic product (13). Considering an essential part of the fight against COVID-19, working from home allowed the lockdown to endure without an ensuing collapse to the economy. As a necessary consequence, the stigma against remote workers has dissipated and many organizations are developing plans to allow for more work-from-home options beyond the duration of the pandemic, with the potential for the number of working days spent at home expected to increase to 20% compared with 5% prior to the pandemic levels (13). Although not available to everyone in all types of work, this shift has yielded enormous changes allowing individuals to save time and money previously spent on commuting. While the longer-term economic and social fallout of these modifications is still to be realized, and stakeholders and participants alike argue that there is indeed something uniquely human lost through digital interactions that may only be provided for through in-person meetings, the advantages are hard to ignore. Further, few would dispute the benefits that the dramatic fall in commuting traffic has provided for the environment. In addition to images in China's biggest cities showing scarcely before seen clean air and blue skies, and the iconic image of New Delhi's India Gate photographed without its usual ghostly polluted haze, studies have demonstrated significant reductions in air pollutants including nitrogen dioxide during the pandemic (14). Correspondingly, similar changes with day-to-day work meetings have opened our eyes to the redundancies and time lost that existed in our previous work schedules allowing for the potential of greater efficiency and work done in a given work week.

On March 17, 2020, the Association of American Medical Colleges recommended the suspension of medical student clinical rotations, with academic institutions migrating curricula to a virtual format to maintain social distancing among students (15), with evidence of similar or improved learning compared to prior years (16). The pandemic has also disrupted medical education for residents and fellows by imposing necessary limitations to in-person meetings forcing learners and educators to adapt to the “new normal” of remote learning. Such challenges can be transformed into opportunities through rapid innovation and exploitation of technological resources to ensure personal safety while maintaining and potentially improving medical education. Technology has already been playing an increasingly important role in teaching core clinical skills as simulation centers and computerized anatomy laboratories have become more prevalent over time (17). The forced adoption of virtual technologies during this pandemic, however, holds the potential to spur an unexpected yet likely beneficial wider embracing of these, and other, tools in the longer term. Various academic organizations have described successful experiences implementing virtual education programs for medical students in diagnostic radiology (18), surgery (19), and other specialties (20). In one published experience, students were exposed to electives in interventional radiology (21) that devised curricula utilizing a combination of synchronous and asynchronous learning and the “flipped” classroom educational model. Synchronous learning is when students and instructors engage in real time, typically utilizing videoconferencing and/or chat software to allow for live interaction, while asynchronous learning refers to learning that occurs at different times for each student, without real-time interaction, making use of resources such as assigned readings or prerecorded videos provided by the instructor (22, 23). A “flipped classroom” model is when students are provided asynchronous educational material to review to establish background knowledge prior to participating in a synchronous lecture on the same topic during which facilitators focus on clarifying concepts, sharing clinical pearls, and engaging with students with virtual lectures (22, 23). In one pilot study, the investigators showed that this “flipped classroom” strategy improved knowledge acquisition with no increase in preparation time and was in fact widely preferred by trainees (24). In another example of the use of virtual technology, while prior to the pandemic medical students and residents attended in-person resident education conferences each morning, during the pandemic these conferences were held virtually to maintain education, a familiar experience in programs across the country. Given that residents and medical students work closely during clinical rotations, residents acting as teachers restored some semblance of normalcy for both groups and allowed residents the chance to refine their teaching skills (25). Key to the evolution of these educational strategies has been the development and sharp increase in the use of commercially available videoconferencing and remote sharing applications such as Zoom (Zoom Video Communications, San Jose, CA, USA), WebX WebEx (Cisco Webex, Milpitas, CA, USA), and Skype (Skype Technologies, Palo Alto, CA, USA). These formats allow trainees and staff to share slides, images, PowerPoint presentations, and other materials remotely while having a live video feed so that each person can see who is present and can engage in dialog in a manner that gives the feel of an in-person meeting. Users can log in from computers but have the flexibility of accessing meetings from smartphones and tablets as well. Other useful benefits include the fact that this format allows administrators to record conferences providing the option for later review, as well as a live chat and even polling functions to add to the learning experience. Such formats can be used to provide educational lectures internally and even to worldwide audiences in an open-access format.

While a return to an in-person education model seemed highly desirable in the early stages of the pandemic, many of the creative changes developed over this time are rightly here to stay having shown that aspects of virtual education are not only possible and of similar value to in-person education but in many ways offer important advantages. The waning of previous resistance to technology-enhanced learning is being increasingly accompanied by evidence of its ability to embellish educational opportunities.

## Vaccine development

A further aspect of the pandemic chapter which must not be overlooked is the development, testing, and mass uptake of multiple effective and safe vaccinations against COVID-19. This impressive feat invoked an unprecedented level of international cooperation and government–private sector collaboration that could in fact form a novel framework for future vaccine development. Vaccinations are one of the world's most efficacious interventions against disease estimated to save 3 million lives each year (26). In 1796, Edward Jenner discovered that exposing individuals to small amounts of the cowpox virus, known as the “vaccine virus,” was effective in preventing smallpox (27). While approximately 300 million people died due to smallpox in the twentieth century alone, the consistent application of global vaccination programs meant that by 1980 the World Health Assembly could officially declare the eradication of smallpox (28). Similar success stories include measles, diphtheria, and rubella whose vaccinations resulted in the >99% decrease in cases in 2019 with respect to the average annualized morbidity in the twentieth century. In fact, one dose of the measles, mumps, and rubella vaccine has an efficacy of 93% against measles, 78% against mumps, and 97% against rubella (29). It must, however, be recalled that for most diseases developing a vaccine takes more than 10 years, as part of an expensive and linear process in which each step is carried out sequentially. Specifically, five stages are involved: (i) discovery laboratory-based research, looking at ways to induce an immunologic response, normally requiring 2–5 years; (ii) preclinical stage, involving testing various compounds in animals to assess safety and appropriateness for use as a potential vaccine in humans, usually requiring 2 years; (iii) clinical development, testing potential vaccines in humans as part of phase I (testing for safety), phase II (further testing for safety, determining suitable dosages, and understanding the immune response), and phase III (assessing the vaccine for efficacy and safety in thousands of patients) trials that typically require 2, 2–3, and 5–10 years, respectively; (iv) regulatory approval, by submitting data to regulatory authorities for review, requiring up to 2 years; and (v) manufacturing and delivery, requiring specialized and expensive facilities. Regulatory authorities continue to monitor safety and efficacy after a vaccine has been licensed and made available. This process is further complicated by the fact that many candidate vaccines never progress beyond the preclinical stage as they fail to produce a desired immune response, fewer than 10% of drugs that enter clinical trials are ever approved by the Food and Drug Administration (30), and a vaccine for a coronavirus has never been developed before. Further, the fastest a vaccine has been developed previously is 4 years, which was against mumps in 1967. Meanwhile, the vaccine against varicella, released in 1995, took 28 years to develop, license, and distribute. While certain steps in the developmental timeline of a vaccine may be fast-tracked or bypassed, the approval step does not fall under this category, and previous incidents in which poorly produced batches of a vaccine that was approved hurriedly leading to individuals contracting and even dying of infections loom large.

Given how deadly and disruptive the pandemic has been, the development of a vaccine against COVID-19 necessitated a radical restructuring to traditional vaccine development. These involved several important adjustments. First, different stages of the development and production of the vaccine occurred at the same time, and multiple vaccine trials were being carried out in parallel around the world. In the United States, three vaccines are currently authorized and recommended, namely, Pfizer-BioNTech, Moderna, and Johnson & Johnson/Janssen. The Pfizer/BioNTech vaccine was the first mRNA vaccine, followed by the Moderna vaccine, to be used in humans outside of clinical trials pioneering mRNA technology to deliver the coronavirus S protein's genetic material into target cells. All vaccines have been evaluated in randomized clinical trials and have been shown to be safe, effective, and capable of reducing the risk of severe illness (31). The Pfizer/BioNTech vaccine has reported the highest efficacy at 95% (32) although it has the disadvantage of requiring storage and shipping at around −70 degree Celsius. Second, multiple vaccine types were funded at the same time using different and often novel technologies providing not only the best chance of finding one that works, but also a diversity of vaccines capable of being effective across different populations. It was estimated that more than 100 vaccines were being developed across the world by June 2020 within exploratory, preclinical, and phase I studies using a broad range of technologies including an inactivated, non-replicating, or replicating viral vector, recombinant protein- or peptide-based vaccines, and viral DNA- or RNA-based vaccines (33). Third, manufacturing was started before vaccines were proven to be safe and effective to avoid delay while incurring significant risk to manufacturers. New manufacturing sites highly tailored to the production of the new vaccines were also built around the world. Fourth, existing technological progress further helped advance the rapid development of the vaccine. Using genomic sequencing, researchers successfully uncovered the viral sequence of COVID-19 by January 2020, 10 days after the first reported case of pneumonia in Wuhan, and the previously studied SARS virus is approximately 80% identical to COVID-19, both of which use the so-called spike protein to grab onto a specific receptor found on cells in human lungs (34). Similarly, early efforts by scientists at Oxford in creating an adenovirus-based vaccine against MERS provided important experimental groundwork in developing an adenovirus vaccine against COVID-19. Last, a new collaborative approach to science and global manufacturing and distribution has been established, without trivializing testing and safety measures, and ensuring the same ethical, scientific, and statistical standards are maintained as in traditional development programs. A study in 2018 estimated the cost of early development and initial clinical safety trials for a typical vaccine to be in the range of $31–68 million (35), which with large-scale trials and an accelerated timetable would likely be an underestimate for COVID-19. Yet, in the United States, Operation Warp Speed partnered with multiple institutions, including the National Institutes of Health and the Centers for Disease Control and Prevention, when developing, manufacturing, and distributing their target of 300 million doses by early 2021. Similarly, the UK government vaccine Taskforce was a significant contributor to a wide variety of vaccine research, with recipients of this funding helping to develop the Oxford/AstraZeneca vaccine (31). The rapid completion of clinical trials was also facilitated by a high interest in volunteers for vaccine studies further highlighting a collaborative spirit. Skeptics argue that the unprecedentedly accelerated timeline in approving and distributing the new vaccines generates legitimate causes for concern. Indeed, the sheer rapidity in the evolution of these vaccines, their approval for use, and the accompanying public health policies that facilitated their mass have been impressive feats, underscoring the benefits of well-organized and collaborative efforts in tackling global health challenges.

An important caveat that must be kept in mind, however, is that while vaccines are the best chance to control the pandemic, these efforts can be thwarted when world leaders succumb to vaccine nationalism. Indeed, vaccine equity is not just a theoretical slogan but above all protects people worldwide from new vaccine-resistant variants. Vaccine nationalism is already setting a foundation for itself and is socially and economically counterproductive, particularly in low-and-middle-income countries (36). We should, therefore, be prepared to enhance awareness of and employ counter measures against this trend to ensure that the success of vaccine development programs may be realized by all.

None of us will miss this pandemic or the trials it has imposed on us. Returning to a life and world glowing with nostalgia sounds appealing, and in many ways it will be. But too much has been sacrificed to the worst yet of this century's global pandemics for us to disregard the benefits and innovation acquired during this time. To quote Churchill again “never was so much owed by so many to so few.” In innumerable ways, the Second World War formed an inflection point that shaped world affairs in ways we can see even today. So too will this pandemic have implications for years to come. The pre-pandemic ways of practicing healthcare, work, and education can be improved upon to create a new and potentially better “normal.” We should be willing to acknowledge and retain useful changes that have been made to our working lives and embrace important lessons in how we collectively tackle our workplace, societal, and public health challenges—unique lessons offered to us from the current crisis.

## Author contributions

The author confirms being the sole contributor of this work and has approved it for publication.

## Conflict of interest

The author declares that the research was conducted in the absence of any commercial or financial relationships that could be construed as a potential conflict of interest.

## Publisher's note

All claims expressed in this article are solely those of the authors and do not necessarily represent those of their affiliated organizations, or those of the publisher, the editors and the reviewers. Any product that may be evaluated in this article, or claim that may be made by its manufacturer, is not guaranteed or endorsed by the publisher.
